# A user-friendly goniometer-compatible fixed-target platform for macromolecular crystallography at synchrotrons

**DOI:** 10.1107/S1600576725011513

**Published:** 2026-02-17

**Authors:** Swagatha Ghosh, Analia Banacore, Per Norder, Monica Bjelčić, Arpitha Kabbinale, Padmini Nileshwar, Gabrielle Wehlander, Daniele de Sanctis, Shibom Basu, Julien Orlans, Adams Vallejos, Leonard M. G. Chavas, Richard Neutze, Gisela Brändén

**Affiliations:** a Department of Chemistry and Molecular Biology Gothenburg University Sweden; b Department of Applied Physics, Graduate School of Engineering Nagoya University Japan; c BioMAX Beamline, MAX IV Laboratory Lund University Sweden; d European Synchrotron Radiation Facility Grenoble France; e European Molecular Biology Laboratory, Grenoble, France; f Nagoya University Synchrotron Radiation Research Center Nagoya University Japan; Uppsala University, Sweden; The European Extreme Light Infrastructure, Czechia

**Keywords:** serial synchrotron X-ray crystallography, fixed-target chips, *in situ* crystallization, room-temperature protein structures, macromolecular crystallography

## Abstract

Fixed-target platforms provide convenient support for microcrystals during serial X-ray crystallography studies using synchrotron radiation. Here, we describe a simple user-friendly 3D-printed support where the crystals are sandwiched between two layers of thin X-ray-transparent membrane resulting in very low scattering background. The platform is compatible with magnetic mounting onto the standard goniometer of macromolecular crystallography beamlines. Our design utilizes a 96-well frame that facilitates hanging-drop experiments directly on the membrane using conventional crystallization plates, thereby eliminating multiple pipetting and crystal handling steps. Crystals can be enclosed in a sandwich and packed into ‘cassettes’, preventing the risk of the sample drying out during room-temperature transportation to synchrotron sources. The versatility of the platform is demonstrated by five structures solved using different crystallization and data-collection strategies. Lysozyme single-crystal rotational crystallography at room temperature is shown, as well as microcrystal serial data collection under cryogenic conditions. On-chip microcrystallization is illustrated by use of a photosynthetic reaction center as an example. Finally, serial crystallography data collection at room temperature from microcrystals of the membrane protein cytochrome *c* oxidase crystallized in lipidic cubic phase is presented.

## Abstract 2

A user-friendly fixed-target platform for serial synchrotron crystallography is described, which allows low-background X-ray diffraction data collection, *in situ* crystallization and transportation of protein microcrystals at room temperature.

## 1. Introduction

X-ray crystallography remains a widely used and well established structural biology technique for obtaining high-resolution atomic-level structures of macromolecules (Brito & Archer, 2020 ▸). A majority of the protein structures deposited with the Protein Data Bank (PDB; https://www.rcsb.org/) emerge from X-ray crystallography, owing to its widespread use and the efficiency of the method where several of the steps can be automated, including the use of crystallization robotics as well as automation of data collection and data processing. Moreover, as the crystals are kept frozen, they can easily be shipped to a synchrotron facility allowing remote data collection. However, conventional X-ray crystallography typically requires protein crystals that are at least tens of micrometres in size, manual crystal handling and cryogenic data-collection conditions to limit radiation damage.

Serial X-ray crystallography (SX) was initially developed at X-ray free-electron laser facilities (XFELs) (Chapman *et al.*, 2011 ▸) and is now established also at synchrotron radiation facilities (Gati *et al.*, 2014 ▸). Serial data-collection methods allow high-resolution room-temperature structural information to be obtained and have expanded the capabilities for time-resolved structural studies to track protein dynamics (Pearson & Mehrabi, 2020 ▸; Brändén & Neutze, 2021 ▸). In SX, X-ray diffraction images are collected from a large number of randomly oriented small crystals where each crystal that is exposed to the X-ray beam gives rise to one diffraction image. Typically, thousands of images are then indexed, integrated and merged to obtain a complete dataset. As each crystal is exposed to a single short X-ray pulse, there is potential to minimize radiation damage. Moreover, combining data from numerous small crystals allows structure determination from micro- and nanocrystals, and may aid in the study of challenging targets like membrane proteins where larger crystals are more difficult to obtain (Weierstall, 2014 ▸). As a hybrid alternative using lower-intensity synchrotron beams, narrow wedges of data can be collected from a smaller set of crystals at cryo- or room temperature (Zander *et al.*, 2015 ▸). The recent development of purpose-built SX synchrotron beamlines with micro-focus X-ray beams and fast detectors is now allowing many more users to access the method.

SX sample delivery is dependent on continuously replacing the crystals and can be broadly categorized into two main branches: moving-target and fixed-target approaches. Moving-target techniques include injection methods that involve a continuous flow of microcrystals across the X-ray beam, *e.g.* by use of a liquid or high-viscosity jet, a capillary, or a microfluidic chip (Weierstall, 2014 ▸; Ghosh *et al.*, 2023 ▸; Monteiro *et al.*, 2020 ▸). Diffraction images are collected as crystals flow across the beam one after the other (Doppler *et al.*, 2022 ▸). This approach enables continuous sample replenishment, allowing data collection from a large number of crystals without manual intervention and at a high repetition rate. It is often the method of choice for collecting time-resolved X-ray diffraction data, where a reaction is initiated in the sample at a selected time-point before the X-ray probe. The majority of time-resolved SX studies to date have been of light-activated proteins, where a laser trigger is used to start the reaction (Brändén & Neutze, 2021 ▸; Khusainov *et al.*, 2024 ▸). A more universal triggering method is by mixing the crystalline sample with a substrate, although the time resolution achieved can be limiting (Monteiro *et al.*, 2020 ▸). A severe limitation of the injection methods is that the sample consumption can be very high, especially for the collection of time-resolved data (Lyubimov *et al.*, 2015 ▸).

An attractive alternative to the injection devices for sample delivery is the use of fixed targets, also called ‘chips’ (Roedig *et al.*, 2015 ▸). Fixed-target approaches involve distributing the crystals on a solid support such as a membrane or mesh that is then mounted on a specialized sample holder and raster-scanned across the X-ray beam. Their main advantages are that they allow minimal sample consumption and that the complexity of the experimental setup may be reduced. Depending on the choice of device, crystals can be either in solution phase or in a high-viscosity medium such as lipidic cubic phase (LCP) (Berntsen *et al.*, 2020 ▸). Potential drawbacks include increased background scattering from the support and, in some cases, the need for precise scanning mechanisms at the beamline. In addition, sample handling during the loading of the chip may cause physical damage to the crystals (Martiel *et al.*, 2019 ▸). Finally, the use of fixed targets can be limiting for time-resolved studies, although there are setups that work very well in combination with both light-triggering (Caramello & Royant, 2024 ▸; Schulz *et al.*, 2022 ▸) and mixing (Mehrabi *et al.*, 2020 ▸). Two conceptually different variants of fixed-target devices have been developed: aperture aligned or sequentially exposed/directed raster (Carrillo *et al.*, 2023 ▸). The aperture-aligned approach relies on a precise location of the crystals on the chip (Owen *et al.*, 2023 ▸). This can be achieved through the use of micro-patterned chips where the crystals arrange themselves in the wells and excess solution is blotted away (Roedig *et al.*, 2016 ▸). One of the earlier examples is the Roadrunner (Roedig *et al.*, 2017 ▸), which utilizes a dedicated sample stage and a humidity chamber to keep the crystals from drying out. The well spacing and pore size are chosen to match the crystal size, but using very small or thin crystals is problematic as they may escape through the pores. A more recent development is the micro-structured polymer (MISP) chip, which allows a cheaper and sturdier alternative (Carrillo *et al.*, 2023 ▸). The sequentially exposed variant of fixed targets does not depend on placing the crystals in specific locations; instead they are randomly distributed on the support. These are typically simple designs where the protein microcrystals are sandwiched between two membranes and data are collected by raster-scanning over a pre-defined grid on the chip (Owen *et al.*, 2017 ▸). An example is the hermetically sealed sheet-on-sheet (SOS) chip, which can be used at both XFELs and synchrotron beamlines (Doak *et al.*, 2018 ▸). To limit the movement of the crystals on the chip during data collection, a variant with 10–15%(*w*/*v*) viscous gelatin and 1–4%(*w*/*v*) agarose gel on the membrane was developed (Lee *et al.*, 2020 ▸). There are also tools available that allow data collection under anaerobic conditions (Rabe *et al.*, 2020 ▸; Bjelčić *et al.*, 2023 ▸). Most purpose-built SX synchrotron beamlines, including MicroMAX at MAX IV (Gonzalez *et al.*, 2025 ▸), ID29 of the ESRF (Orlans *et al.*, 2025 ▸), T-REXX of Petra III and I24 of Diamond, offer access to various fixed-target devices, some of which are summarized in Table 1 ▸ with the characteristics of each device indicated.

One of the previous major limitations with the SX method was the challenge of producing suitable and sufficient amounts of microcrystals. Batch crystallization with seeding has proven to be an efficient method for proteins in solution (Dods *et al.*, 2017 ▸; Dunge *et al.*, 2024 ▸; Shoeman *et al.*, 2023 ▸), and approaches for large-scale LCP crystallization of membrane proteins have been presented (Andersson *et al.*, 2019 ▸). A risk associated with crystallization is that of damaging crystals during handling. Therefore, it may be advantageous to integrate crystallization with data collection, for example through *in situ* crystallization where the crystals are directly grown on the support that is used for data collection (Foos *et al.*, 2024 ▸). Some crystallization plates allow direct data collection at room temperature, where typically a wedge of data is obtained through a small rotation of the plate in the beam (Axford *et al.*, 2012 ▸; Lieske *et al.*, 2019 ▸; Thompson *et al.*, 2024 ▸), and specialized plates enable the collection of diffraction data also under cryo-conditions (Broecker *et al.*, 2018 ▸). *In situ* crystallization to screen for suitable crystallization conditions has also been achieved in microfluidics chips (Sui, 2017 ▸). However, *in situ* crystallization is not routinely performed in combination with SX fixed-target devices.

Despite the rapid developments over the past decade, many challenges for making the method easily accessible to new users remain. There are issues associated with transportation of crystals at room temperature, which means that crystals in many cases have to be prepared on-site at the radiation facilities. Data-collection devices need to be more user friendly and affordable. Finally, SX data collection would benefit tremendously from the use of robotics to automatically mount the fixed targets at the beamline. In this work, we present a flexible and easy-to-use 3D-printed fixed-target platform. It allows on-chip crystallization, can be transported safely, is pre-assembled to aid sample loading, is compatible with data collection at all common synchrotron beamlines without specialized hardware, and gives high-quality SX data at room temperature as well as under cryo-conditions. The versatility of the device is showcased by presenting structural data collected at three different synchrotron facilities on three protein systems including a membrane protein crystallized in LCP.

## 2. Materials and methods

### 2.1. Design of a chip-based framework for hanging-drop crystallization, crystal transportation and data collection

**Figure 1:**
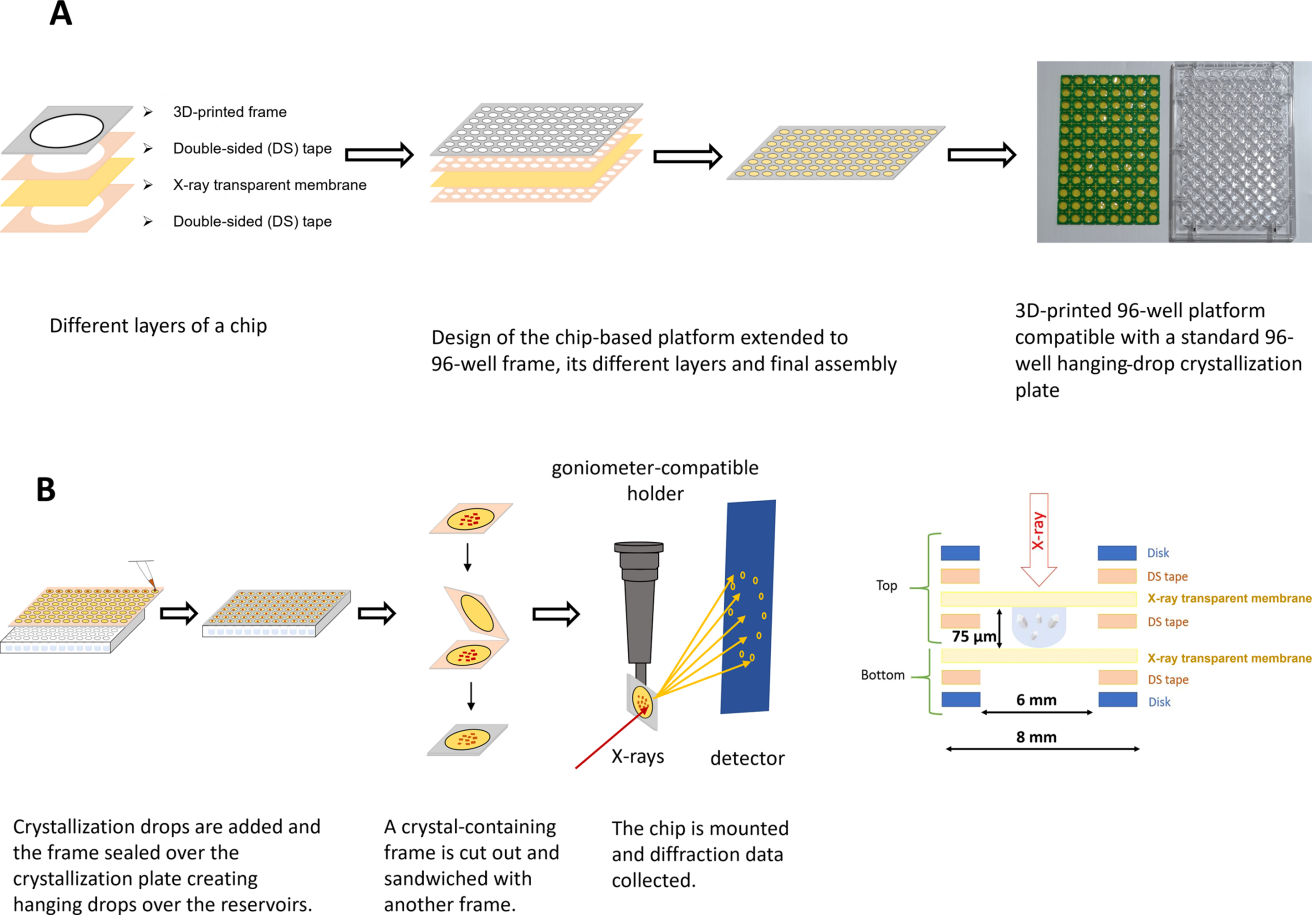
Design and use of the fixed-target device. (A) Design, development and fabrication of the platform consisting of a 3D-printed frame (with disk), which is assembled with double-sided tape, and an X-ray-transparent membrane. (B) Illustration showing the use of the platform for high-throughput hanging-drop crystallization, assembly of a crystal-containing chip and sample delivery in front of the X-ray beam using a goniometer-compatible holder for diffraction data collection. The design results in a path length of 75 µm for the X-ray beam through the crystals in the enclosed chip.

**Figure 2:**
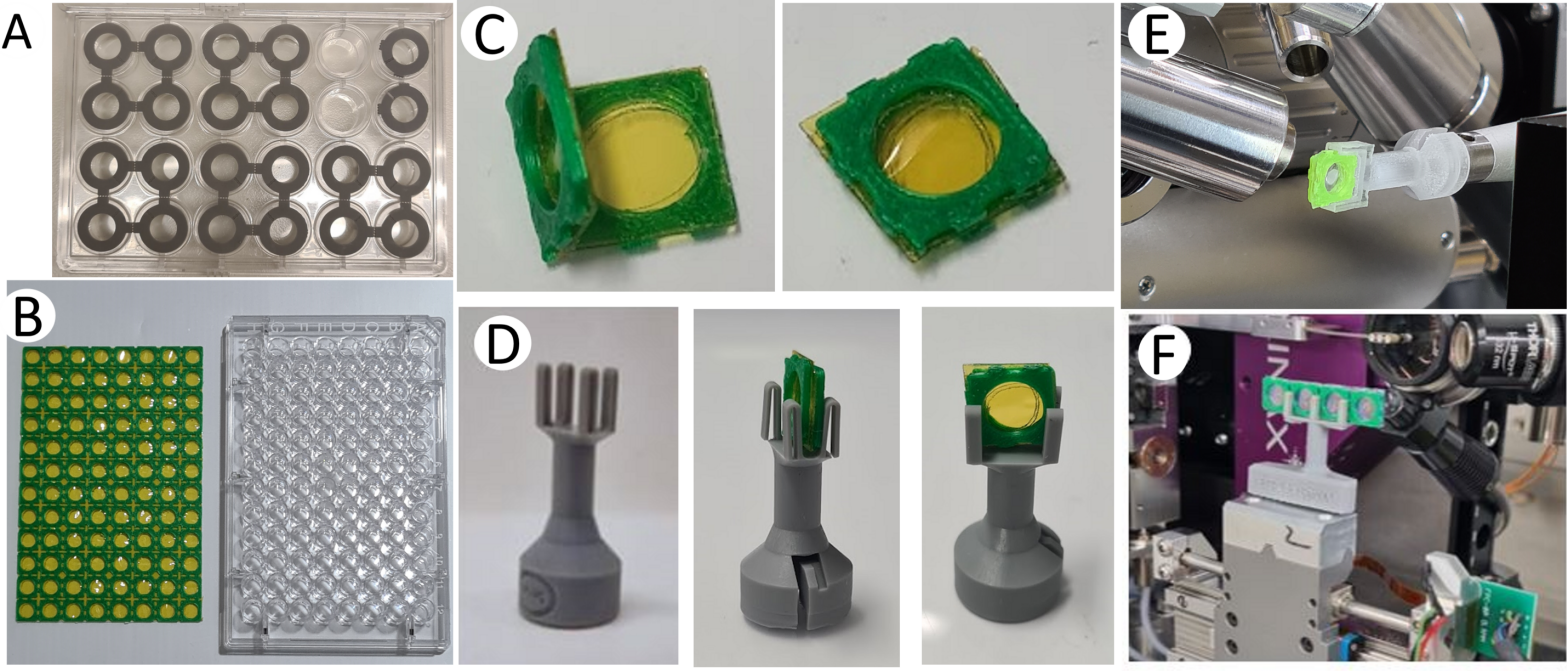
Design and assembly of the chip. (A) The 24-well design of the chip. (B) The 96-well design with a top frame next to a crystallization plate. (C) Chip assembly. (D) The chip holder with and without the chip inserted. (E) The chip mounted on the goniometer. (F) Multiple chips mounted on the goniometer.

**Figure 3:**
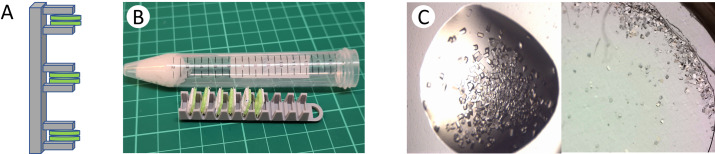
Shipment and storage of crystal-containing chips. (A) Cassette (gray) for storage and transportation of the assembled sandwiched chips (green). (B) Once the chips are clipped onto the holder, the entity is loaded into a plastic tube containing a moist cotton pad/paper soaked with crystallization solution to prevent the samples from drying out. A 3D-printed holder for the 96-well sandwiched chips compatible with a 15 mL plastic tube is shown. (C) Crystals of lysozyme grown on a chip composed of a 3.6 µm Mylar membrane were captured before (left) and after (right) shipment of the sandwiched chips from the home laboratory (Gothenburg, Sweden) to the ID29 beamline of ESRF (Grenoble, France) with a total duration of ∼7 days.

The chip-based platform described here consists of three parts: (1) a framework composed of ‘chips’ for hanging-drop crystallization and/or encapsulation of crystals, (2) a compact device for crystal transportation, and (3) a goniometer-compatible holder for X-ray diffraction data collection. The models for different parts of the platform were prepared using computer-aided design technology with *AutoCAD* (https://www.autodesk.com) and printed in our in-house 3D printers (Asiga MAX/MAX UV) using plastic resin (PlasGRAYV2) and/or FELIX Pro 3 printer (with polylactic acid, PLA filaments). Firstly, 3D models of a solid frame containing a circular disk (or hole) that could fit onto a reservoir of a standard 24-well (Hampton Research) or a 96-well (Greiner and TPP) hanging-drop crystallization plate were printed. Each disk was then layered with an X-ray-transparent membrane composed of Mylar (3.6 or 6.0 µm thickness) or Kapton (7.6 or 12.7 µm thickness) using an adhesive. Double-sided adhesive films accurately fitting the size of the disks were carved on a Silhouette cameo-4 cutting machine. The 3D-printed frame with disks, double-sided adhesive films and an X-ray-transparent membrane was assembled as shown in Fig. 1 ▸A into an entity called a ‘chip’. For ease of assembly, the 96-well design makes up an array of chips that could fit over a standard crystallization plate. Post-assembly, the frames were wiped with ethanol and dried before being used for crystallization or data collection. Crystalline samples are mounted by closing two chips to form a sandwich (Figs. 1 ▸B and 2C) with a total thickness of ∼75 µm that is intercepted by the X-rays, or 150 µm if two spacers are used. For storage and transportation, different versions of a 3D-printed stacking device called a ‘cassette’ were developed that are compatible with the 24-well and 96-well formats of the chips (Fig. 3). The cassettes can be readily carried in 15 ml (96-well format chip) or 50 ml (24-well format chip) plastic tubes, allowing a portable way to hold ten sandwiched chips for storage and transportation of crystals to the synchrotron at room temperature. Lastly, to facilitate mounting of the chips onto a beamline goniometer for diffraction data collection, a holder based on a previously reported goniometer-compatible flow-cell device was fabricated (Ghosh *et al.*, 2023 ▸). Briefly, the 3D-printed nozzle of the flow-cell was altered and equipped with grooves to hold the chips tailored for various chip sizes and arrays of chips (Fig. 2D). During data collection, a magnet is inserted at the base of the holder. The holder is mounted on the goniometer in the experimental hutch of the beamline and operated using standard software (Fig. 2E and F).

### 2.2. Preparation of protein crystal samples

*Lysozyme*. For the room-temperature structure, lysozyme was crystallized as reported previously (Diamond, 1974 ▸). Briefly, lysozyme from hen egg white was purchased from WAKO Chemical Corporation (Lot PDN2655) and dissolved in 0.05 *M* sodium acetate buffer (pH 4.5) to a final concentration of 80 mg mL^−1^. The solution was filtered through a 0.22 µm filter to remove particulates. Using a 96-well plate, 100 µL of the crystallization solution was pipetted into the reservoir wells to obtain a concentration of 0.1 *M* Na acetate (pH 4.5) and 1–2.5 *M* NaCl. A 1.5 µL drop of 1:1 mixture of lysozyme and reservoir solution was placed on each of the chips containing a 12.7 µm Kapton membrane, after which the chip was flipped and pressed over the well of the plate. Crystals of lysozyme grew to 150–300 µm (Fig. 5A) in size after overnight incubation at 20 °C.

For the cryogenic structure, chicken egg white lysozyme (Sigma, CAS-12650–88-3) was dissolved at a concentration of 50 mg mL^−1^ in 0.1 *M* Na acetate (pH 3.0). A 500 µL portion of this solution of was mixed with 500 µL of precipitant consisting of 17% NaCl, 5% PEG 8000 and 0.06 *M* Na acetate (pH 3.0) in an Eppendorf tube and incubated overnight at 20 °C. The resulting crystal pellet (10 µL) was diluted with 20 µL of the precipitant buffer, with subsequent addition of 3 µL of 50% glycerol.

*Photosynthetic reaction center from Blastochloris viridis (RC_vir_)*. The growth, purification and crystallization of RC*_vir_* were described previously (Dods *et al.*, 2017 ▸). *B. viridis* cells were grown in an anaerobic environment for 36 h in the dark and 48 h in the light. Cells were harvested, disrupted by sonication and centrifuged to isolate membranes containing RC_*vir*_. Membranes were solubilized overnight in Tris buffer containing lauryldimethyl­amine-*N*-oxide detergent and centrifuged. The protein was purified using anion exchange chromatography and gel filtration. For crystallization, a protein concentration of 7.5 mg mL^−1^ was used together with a crystallization solution (400 µL of heptanetriol, 20 µL of 1 *M* KPi pH 6.8 and 475 mg of ammonium sulfate) and crystal seeds. Two methods of crystallization were utilized (Fig. 5B). Firstly, RC*_vir_* crystals were grown in sitting drops at 4 °C and 3–5 µL of crystal slurry was pipetted onto the chip with a 12.7 µm Kapton membrane. Alternatively, microcrystals were generated at 4 °C directly onto a chip with a 3.6 µm Mylar membrane using the hanging-drop method with additional 2 *M* ammonium sulfate in the reservoir.

*ba_3_-type cytochrome c oxidase from Thermus thermophilus (Tt CcO)*. Microcrystallization of *Tt* C*c*O in LCP was performed by modifying the previously reported method (Andersson *et al.*, 2017 ▸). Briefly, *Tt* C*c*O was extracted and purified from bacterial membranes using affinity purification with Ni–nitrilo­tri­acetic acid followed by overnight dialysis at 4 °C and ion-exchange chromatography using a HiPrep DEAE FF 16/10 column. Purified protein was concentrated to 12–15 mg mL^−1^ and crystallized in LCP using a well-based technique in glass plates at room temperature as described previously (Andersson *et al.*, 2019 ▸). The crystallization buffer contained 0.1 *M* MES (pH 5.3), 1.4 *M* NaCl and 39–41%(*v*/*v*) PEG 400. The slightly higher PEG 400 concentration in our sample compared with that used in previous work led to partial melting of the LCP during crystallization and resulted in crystals of slightly larger size, measuring 35–40 µm in their longest dimension. The crystal sample was harvested after 2–3 days, packed into a PCR tube and carried to the synchrotron.

### 2.3. X-ray diffraction data collection, processing and structure determination

On-chip-grown lysozyme crystals were sandwiched and mounted, after which data collection on single crystals was performed at room temperature at the Photon Factory (PF) (Hiraki *et al.*, 2008 ▸) Advanced Ring (AR) AR-NW12A beamline (Chavas *et al.*, 2012 ▸) of Japan’s High Energy Research Organization (KEK) radiation facilities. The beamline control software *UGUI* was used for sample viewing, alignment and diffraction measurements. We utilized an X-ray beam of 200 µm (V) × 130 µm (H) at 0.75 Å wavelength, an exposure time of 0.1 s per frame, a flux of 5 × 10^11^ photons s^−1^ and 100% transmission. Diffraction data were collected on a PILATUS3 S2M detector while rotating the chips from 50° to 130° in reference to the chip surface with 0.1° of oscillation. The data were auto-processed using scripts adapted within the in-line PREMO (PF Remote Monitoring) system to generate MTZ files. The MTZ files were truncated and molecular replacement with PDB model 3wun (M. Sugahara, E. Nango & M. Suzuki, to be published) using *Phaser* (McCoy *et al.*, 2007 ▸) in *CCP4i* (version 8.0.010) (Winn, 2003 ▸) was employed. The structure was refined in *CCP4i* as well as using the *PHENIX* suite (version 1.17.1-3660) (Adams *et al.*, 2010 ▸).

Serial synchrotron crystallography on lysozyme, *Tt* C*c*O and RC*_vir_* was performed at the BioMAX beamline of MAX IV Laboratory (Sweden) (Ursby *et al.*, 2020 ▸) and the ID29 beamline of ESRF (France) (de Sanctis *et al.*, 2012 ▸; Orlans *et al.*, 2025 ▸). Lysozyme crystal slurry (1.5 µL) was added to a 6.0 µm Mylar chip and frozen directly in the cryo-stream before data collection. In the case of C*c*O and RC*_vir_*, 2 µL of microcrystals were sandwiched on chips with a 12.7 µm Kapton membrane (Fig. 5C), which were mounted on a holder and aligned with the X-ray beam for data collection at room temperature. Crystals were viewed on *MxCuBE3* (Mueller *et al.*, 2017 ▸) and appropriately sized grids were selected for data collection. The data were collected using the standard grid scan of the beamlines where the separation between grid points is defined by the size of the X-ray beam. The lysozyme data at cryogenic temperature were collected at BioMAX with an X-ray beam size of 20 µm (V) × 5 µm (H), a wavelength of 0.7293 Å (or photon energy of 17 keV) and a flux of 1.5 × 10^12^ photons s^−1^, 100% transmission, and recorded on an EIGER 16M CdTe detector at a frame rate of 10 ms per frame. At BioMAX, data collection on both C*c*O and RC*_vir_* was performed with an X-ray beam size of 20 µm (V) × 5 µm (H), wavelength 0.98 Å (or photon energy of 12.7 keV) and a flux of 3.81–3.88 × 10^12^ photons s^−1^, 100% transmission, using an EIGER 16M hybrid pixel detector at a frame rate of 10 ms per frame. Finally, at ESRF, an X-ray beam size of 4 µm (V) × 2 µm (H), a photon energy of 11.56 keV, a flux of 2 × 10^15^ photons s^−1^, a frequency of 231.25 Hz with a pulsed beam of 90 ms and a Jungfrau 4M detector was used to collect the second RC*_vir_* dataset. *CrystFEL* (version 0.9.0 and above) (White *et al.*, 2012 ▸; White *et al.*, 2016 ▸) integrated within the pipelines of the beamlines was used for indexing, integration, merging and conversion to MTZ format. The MTZ files were truncated, and the structures were solved by molecular replacement using *Phaser* and refined with the *CCP4i* (Agirre *et al.*, 2023 ▸) and *CCP4* cloud (Krissinel *et al.*, 2022 ▸) suite. Previously known crystal structures with PDB IDs 5nj4 (Dods *et al.*, 2017 ▸) and 5ndc (Andersson *et al.*, 2017 ▸) were used as models of RC*_vir_* and *Tt* C*c*O, respectively.

For all datasets, model building was performed in *COOT* (Emsley & Cowtan, 2004 ▸) and structural illustrations were drawn in *PyMOL* (DeLano, 2002 ▸). Final statistics of all the datasets are presented in Table 2 ▸.

## 3. Results and discussion

Throughout this work, we aimed to simplify the steps required for SX data collection at synchrotron facilities by the development of a fixed-target platform that is pre-assembled, is convenient to use, can facilitate shipment of samples at room temperature and can be mounted upon a standard SPINE magnet for data collection. In this manner, any X-ray diffraction beamline with a rapid-readout X-ray detector could be adapted for SX data collection by making use of the raster-scanning capability of the goniometer. We demonstrate the versatility of this chip-based platform by applying various data-collection strategies at several synchrotron radiation sources targeting different proteins.

### 3.1. Assembly of the chip and hanging-drop crystallization

To minimize crystal handling, we developed a platform that could support hanging-drop crystallization experiments above a crystallization well of a standard crystallization plate, and where the same frame could be used as the mount for X-ray diffraction data collection. We first explored this concept using a 24-well format (Fig. 2 ▸A) and then extended the concept to the 96-well format (Greiner and TPP) plate for hanging-drop crystallization setups.

For the 96-well design, a frame consisting of an array of flat circular supports (Fig. 2 ▸B) was assembled with a layer of X-ray-transparent membrane using double-sided adhesive tape. The thickness of the 3D-printed support was chosen for its light weight and flexibility, and it could be easily detached with a scalpel for assembly of the platform. Our assembly consists of two types of frames: the top frame has a ‘support–adhesive–membrane–adhesive’ design that is suitable for on-chip crystallization, while the bottom frame has only one layer of double-sided tape (‘support–adhesive–membrane’) to be used for encapsulating pre-grown crystals within a sandwich to prevent the crystals from drying out. Two top frames that both contain a layer of adhesive tape can be used to create a chip for data collection that provides larger spacing between the membranes if needed and was used in this study for the high-viscosity *Tt* C*c*O sample crystallized in LCP. Four different variants of X-ray-transparent membranes were explored during development of the platform to find the optimal material: Mylar of 3.6 or 6.0 µm thickness and Kapton of 7.6 or 12.7 µm thickness. Although different membranes have been used to collect the data presented in this study, the 12.7 µm-thick Kapton membrane is recommended for most applications at room temperature as it is damaged less easily and does not wrinkle during the assembly process. For cryogenic conditions, the 6 µm Mylar membrane is superior. The 3D-printed backbone support with adhesive fits onto a 96-well plate, and the double-sided tape successfully creates an environment for vapor diffusion. This design thus supports *in situ* crystallization, and the double-sided tape ensures a closed environment, with no grease being required to seal the chambers. The membrane maintains a humid environment for a crystallization drop of ∼1.5 µL when using a 100 µL reservoir, and there was no indication of drops drying out after three weeks upon visual inspection. Crystal nucleation and growth on the chips can be directly monitored using a light microscope. Once crystals appear, the entire 96-well frame is detached from the crystallization plate using a scalpel and sandwiched with a complementary frame to encapsulate crystals between the two membrane layers, which also prevents the samples from drying out after assembly (Fig. 2 ▸C). Alternatively, a single crystallization drop can be extracted from the crystallization setup by cutting out the 3D-printed plastic frame of one well and sealing it with a complementary frame. Another variant is to leave every second well empty during crystallization, to be able to cut out two adjacent wells at a time and fold them onto each other so as to sandwich the crystallization drop (Fig. 2 ▸C). This alternative leads to a larger spacing between the membranes. This supported sandwich is then clipped onto a holder that is compatible with a SPINE base (Fig. 2 ▸D) and data are collected using a 2D raster scan or by rotation of the chip (Fig. 2 ▸E and F).

The design is also available in a 24-well format. However, the 96-well framework is advantageous for several reasons. As it matches standard 96-well crystallization plates (Greiner and TPP) it is compatible with multi-channel pipettes and sample-dispensing robotics, and the device could potentially be used for high-throughput crystallization screening. Moreover, the 3D-printed support contains perforations between the individual squares that allow the frame to be separated into different parts so that one frame can be used to screen several different proteins or crystallization conditions. During crystallization on the 96-well frame, it is suggested to use a humidifier for sensitive proteins or buffers containing volatile additives to avoid drying of sample during crystallization.

Importantly, the platform also allows the mounting of crystals that have been grown using other methods (Uwangue *et al.*, 2025 ▸). In this case, typically ∼1.5 µL of microcrystals are harvested from a crystallization plate or tube and pipetted directly onto the membrane of the support. In the case of LCP-grown crystals, they are extruded onto the membrane from a Hamilton syringe. An adjacent single frame can then be folded onto the crystal drop to seal it closed. Alternatively, all 96 wells are loaded with crystals and another 96-well support is used to sandwich all 96 drops in one step. In the latter case, once sealed, the sandwiches can be cut out individually and clipped on a holder, which in turn is mounted on the goniometer (Fig. 2 ▸D and E). The procedure is gentle and no signs of crystal fragmentation due to mechanical stress are visually observed upon assembly of the chips. While the mounting may initially seem to involve multiple steps, the procedure requires minimal user training and significantly reduces the risks of crystals being lost or becoming dehydrated during assembly or data collection. To assemble the chips, the only tool that is required is a scalpel to cut out the frames.

In short, the presented fixed-target platform offers several advantages over existing methods (Table 1 ▸). For example, a similar sheet-on-sheet design approach referred to as the SOS chip needs a custom-designed holder to load the crystals, stretch the membrane and seal the device inside a humidified chamber. In contrast, our setup does not require specialized expertise or hardware, it avoids the problem of wrinkles on the membrane, and crystals can be pre-mounted on the chip in the home laboratory for transportation.

### 3.2. Crystal storage and transportation

To store and transport the crystal-containing sandwiched chips at room temperature, we constructed a 3D-printed compact device consisting of a stack of chip holders or ‘cassette’. This cassette is fitted inside a 15 mL Falcon plastic tube (Fig. 3 ▸A and B), a cotton pad soaked with crystallization solution or buffer is added to the tube to retain humidity around the crystals during storage and transportation, and the tube is sealed tightly. Lysozyme crystals shipped in the storage device did not dry out during shipment according to visual inspection (Fig. 3 ▸C), and transported crystals of *in situ* grown RC*_vir_* retained diffraction quality after transportation in the storage system.

### 3.3. Mounting of the chip for data collection

To mount the chip on the goniometer of a standard macromolecular crystallography synchrotron beamline, we adapted the design of a goniometer-compatible flow-cell (Ghosh *et al.*, 2023 ▸) to be able to hold the sandwiched chips for alignment and data collection (Fig. 1 ▸B). More specifically, the 3D-printed part was modified to hold various chip sizes and arrays of chips (Fig. 2 ▸E and F). The height of the holder is compatible with the SPINE standard (Beteva *et al.*, 2006 ▸) to prevent any perturbation of the beamline optics, and the mounting system can easily be adapted to the specific needs of the beamline. For data collection, the sandwiched chips, either assembled at the beamline or transported in the cassette, are slotted into the groove of the holder and mounted onto the goniometer using a magnetic disk at the base of the holder. At some beamlines it is possible for the scan domain of the goniometer to mount an array of chips, which allows a more efficient data collection (Fig. 2 ▸F).

### 3.4. X-ray diffraction data collection and resulting structures

**Figure 4:**
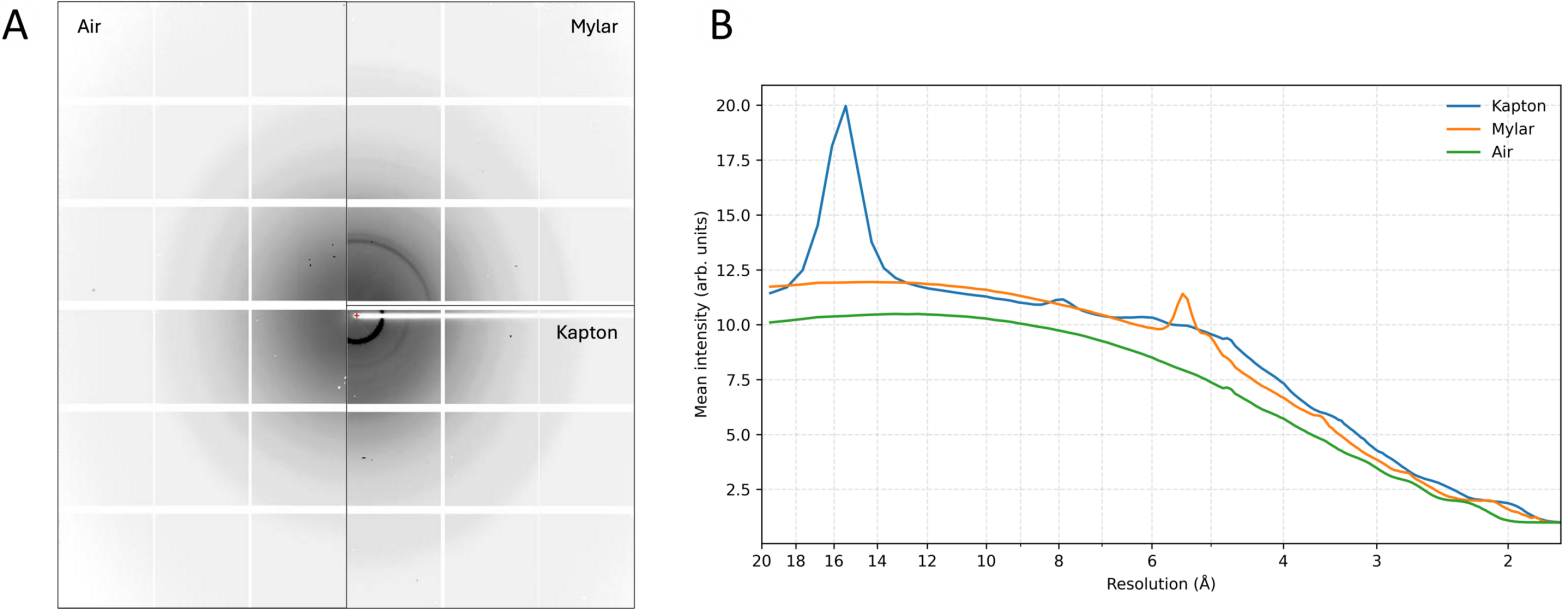
X-ray scattering background of the fixed-target supports. (A) Average of 100 images collected from chips with Mylar (6 µm) and Kapton (12.7 µm) membranes as well as in air for comparison. The data were collected at the MicroMAX beamline at MAX IV, Lund, using an energy of 12.7 keV, a flux of 5.3 × 10^12^ photons s^−1^ and a 10 ms exposure time. (B) The graphs show the mean intensity by resolution (binned into 200 resolution bins) for the averaged images in (A).

To evaluate the scattering background from the device itself, we collected X-ray scattering data in air as well as on two variants of the chip composed of Kapton or Mylar membrane without any sample loaded (Fig. 4 ▸). This confirms the low scattering background of the platform. We then tested the versatility of our fixed-target platform for various X-ray diffraction data-collection strategies at room and cryo-temperature and on crystals from three types of proteins, out of which one is crystallized in LCP. The results are summarized in Table 2 ▸.

**Figure 5:**
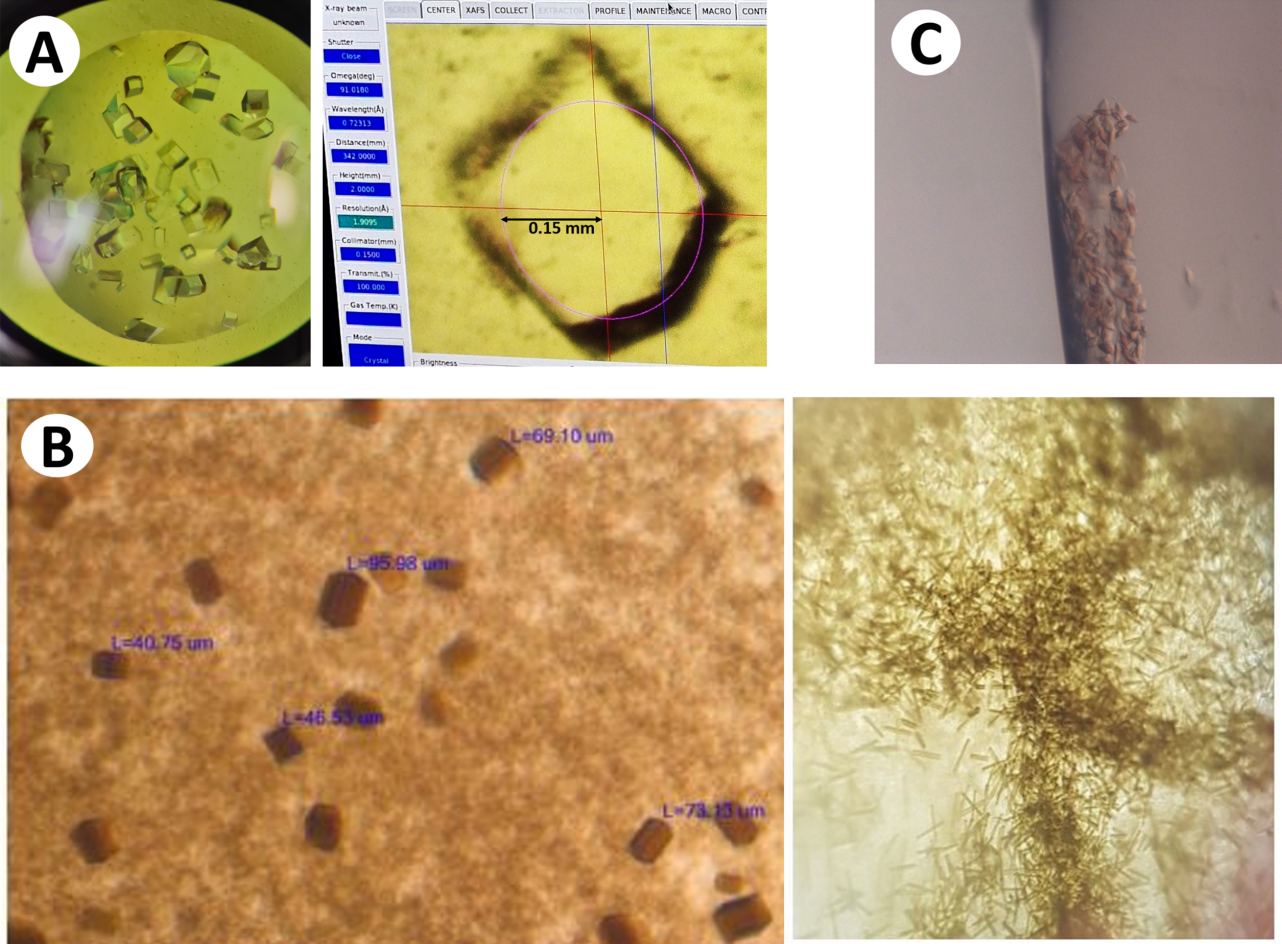
Crystals used for data collection. (A) Crystals of lysozyme of ∼150 × 200 × 200 µm^3^ in size (estimate based on the size of the X-ray beam) in a hanging drop visualized on a 96-well chip (left). A single crystal is selected for data collection at the PF AR-NW12A beamline, Japan (right). (B) Crystals of RC*_vir_* approximately 20 × 40 × 80 µm^3^ in size obtained from on-chip crystallization by the hanging-drop technique on a 24-well chip (left). Crystals of the same protein obtained in a sitting-drop plate with a size of ∼20 × 20 × 100 µm^3^ (right). (C) LCP crystals of *Tt* C*c*O visualized on the chip.

**Figure 6:**
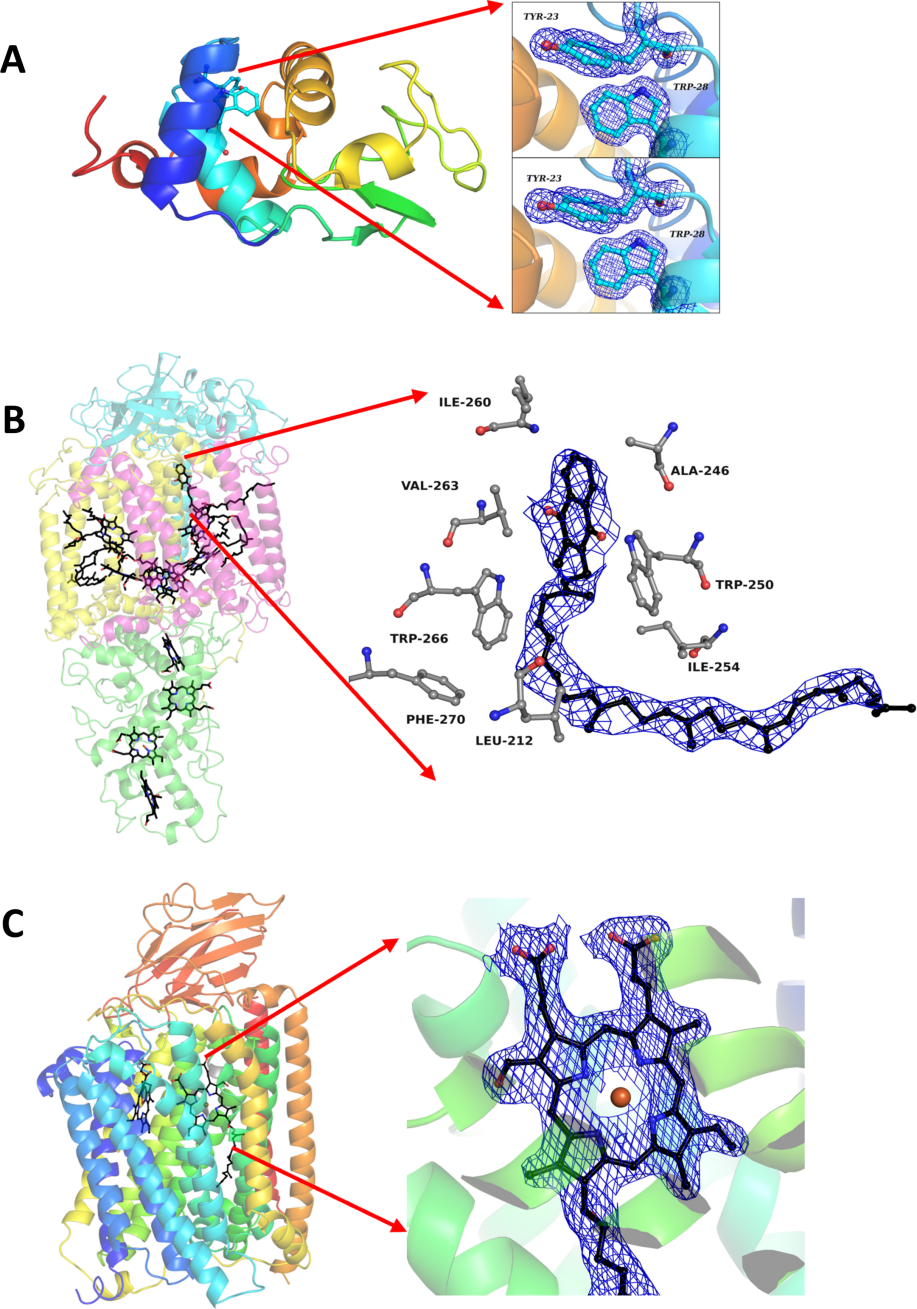
Structures of lysozyme, RC*_vir_* and *Tt* C*c*O. (A) Crystal structures of lysozyme from microcrystals at cryogenic temperature (top) and an *in situ* grown single crystal at room temperature (bottom). 2Fo–Fc (blue, 1.0σ) electron density maps of selected residues are shown. Serial synchrotron crystallography structures of (B) RC*_vir_* and (C) *Tt* C*c*O with a co-factor highlighted in each case. The 2Fo–Fc electron density is contoured at 1.0σ (blue).

The first example is with the model protein lysozyme. Using rotational crystallography, we collected data at room temperature from on-chip-grown single crystals of lysozyme prepared on-site one day before data collection at the PF. Each chip contained several crystals of 150 µm × 200 µm × 200 µm in size (Fig. 5 ▸A), and one crystal was selected for rotational data collection. We noticed a slight movement of crystals enclosed within the sandwiched chip when mounted on the goniometer. This was resolved by removing the excess volume of crystallization solution from the sandwiched chip prior to data collection using a thin capillary wire. During data collection, an opaque edge near the frame of the chips prevented data collection from a complete 360° rotation of the chip. This problem is inherent to the device and can be overcome by collecting data from several crystals that are oriented differently on the chip. For lysozyme crystallized in a high-symmetry space group, an ∼80° rotation of a single crystal was sufficient to obtain a nearly complete dataset. The data at cryogenic temperature were collected on lysozyme microcrystals prepared with the batch method and complemented with the cryoprotectant glycerol before being loaded onto the chip and frozen in the cryo-stream at the beamline. The resulting lysozyme structures were refined to resolutions of 1.6 Å (Fig. 6 ▸A) and 1.75 Å for room and cryo-temperature, respectively.

Crystals of RC*_vir_* grown directly on the membrane surface of the chip differ in morphology and size compared with crystals obtained by the sitting-drop crystallization method (Fig. 5 ▸B). The crystals obtained through on-chip crystallization were larger in size, and a complete dataset was collected using the serial method at the BioMAX beamline of MAX IV Laboratory, resulting in a 2.8 Å structure (Fig. 6 ▸B). For comparison, RC*_vir_* microcrystals grown using sitting-drop vapor diffusion were applied to the 96-well plate frame for data collection at the ID29 beamline of ESRF using a raster scan with a mesh chosen according to the maximum area that the frame allowed. The RC*_vir_* structure solved from sitting-drop microcrystals was refined to a resolution of 3.0 Å. Overall, the structures are in very good agreement with previously solved structures of the RC*_vir_* protein. Our data also agree with the fact that RC*_vir_* microcrystals display weaker diffraction at synchrotron sources than at XFELs, where LCLS data previously resulted in a 2.4 Å resolution structure (Dods *et al.*, 2017 ▸).

LCP-grown crystals of *Tt* C*c*O were pipetted onto the membrane and the frame was sandwiched with an adjacent frame. As both frames contained the adhesive tape, this created a larger spacing between the membranes. The high-viscosity sample was efficiently spread out over the surface as the chip was sealed by gently pressing the two frames together (Fig. 5 ▸C). The volume of LCP applied to the chip in this case was approximately two microlitres, but the volume may be altered depending on the viscosity of the sample. To obtain the maximum number of images from one single chip, several grids can be drawn to cover the surface as efficiently as possible. The *Tt* C*c*O data presented here were collected from three chips where one to three grids were raster-scanned per chip. The resulting structure (Fig. 6 ▸C) at 2.3 Å resolution agrees well with and is of similar resolution to a previously solved structure of *Tt* C*c*O where data were collected using a capillary-based flow-cell (Ghosh *et al.*, 2023 ▸).

From these results it is clear that our chip-based platform allows *in situ* data collection from on-chip-grown crystals, is suitable for single-crystal rotational as well as serial data collection, and is compatible with both room- and cryo-temperature experiments.

## 4. Comparison with other available chip options

A wide variety of alternatives are available for users interested in performing SX data collections using fixed-target devices, including some commercially accessible alternatives. In Table 1 ▸, a selection of these are listed in addition to the fixed-target chip presented in this work. The selected variants display differences regarding which data collection strategies they allow for, if they are compatible with on-chip crystallization or not, and whether they are compatible also with the collection of cryo-temperature data. Out of the eleven options presented, five require specialized hardware or stages to mount the sample onto the goniometer at the beamline. The other six options, including our device, instead take advantage of the SPINE system that is readily available at most macromolecular crystallography beamlines. This alternative makes the setup straightforward and more suitable for non-expert users. A second measure of convenience is the possibility of crystallization directly on the device, *i.e. in situ* data collection without transfer of the crystals. In this case, seven of the listed devices offer this possibility, including our chip. *In situ* data collection can save time and may aid in cases where the crystals are fragile and sensitive to handling. Another property that is listed is whether the device is single use or not. There are pros and cons regarding using a disposable device. The advantage is that a disposable device minimizes the risk of cross-contamination between samples and eliminates the need for time-consuming cleaning procedures, improving efficiency. In contrast, single-use devices generate more waste. Most of the devices listed rely on the raster-scan data-collection strategy, yet some of the devices presented, including ours, are also compatible with rotational data collection. Which of these strategies is most suitable depends on many factors, such as the nature of the sample including crystal size and sample consistency. An issue that was observed in this study is that crystals contained in liquid on some occasions moved within the chip during data collection. This problem was to a large extent mitigated by use of the smaller-size 96-well instead of the 24-well chip and optimization of the crystal sample volume applied to each chip. For some of the devices shown in Table 1 ▸ this issue can be resolved by using a specialized loading system, although this might complicate the sample preparation process. Finally, the chip presented here is compatible with LCP crystals and allowed a high-quality structure of the *Tt* C*c*O membrane protein to be solved.

## 5. Conclusion

In this study, we present a fixed-target device developed for serial crystallography data collection at synchrotron radiation sources. The chip is easy to use, is less fragile than many alternatives, allows on-chip crystallization and enables high-quality X-ray diffraction data to be collected by use of the SPINE-based system available at most relevant beamlines. Importantly, it enables diffraction data to be collected from very low amounts of crystal sample. The benefits of the chip are presented by showcasing results from three different protein systems including a membrane protein crystallized in LCP. Data were collected using different strategies at both cryo- and room temperature to verify the versatility of the device. Finally, a simple system to transport the pre-loaded chips at room temperature and with retained humidity is presented.

## Supplementary Material

PDB reference: lysozyme, 9tbl

PDB reference: lysozyme, 9uyr

PDB reference: photosynthetic reaction center from *Blastochloris viridis*, 9vdx

PDB reference: photosynthetic reaction center from *Blastochloris viridis*, 9s39

PDB reference: cytochrome *c* oxidase from *Thermus thermophilus*, 9vdj

## Figures and Tables

**Table 1:** Examples of fixed-target devices and how they are typically used ‘Base’ refers to whether the fixed target requires a customized base or if it is compatible with the standard SPINE base for data collection (Beteva *et al.*, 2006 ▸). ‘*In situ*’ refers to whether it is suitable for *in situ* crystallization. ‘Single use’ is whether the device excluding the holder/base is disposable, with the advantage of avoiding time-consuming cleaning and the risk of contamination, or reusable, which generates less waste. ‘Collection strategy’ describes if X-ray data are collected using a raster scan or if the crystals are in pre-defined positions (‘aperture aligned’). ‘RT’ and ‘cryo-temp’ refer to whether the data can be collected at room temperature and cryo-temperature, respectively, and ‘sample-loading system’ whether a custom-made system is needed to load the sample onto the chip. The fixed-target device described in this study is highlighted in bold.

Common name	Base	*In situ*	Single use	Collection strategy	RT	Cryo-temp	Sample-loading system	Reference
Oxford	Customized	No	No	Aperture aligned	Yes	No	Yes	Mueller *et al.* (2015 ▸)
Suna	SPINE	Yes	No	Raster	Yes	Yes	No	Roedig *et al.* (2015 ▸)
Diffrax	SPINE	Yes	Yes	Raster	Yes	Yes	No	Axford *et al.* ( 2016 ▸)
Roadrunner	Customized	Yes	No	Raster	Yes	Yes	Yes	Roedig *et al.* (2017 ▸)
SOS	Customized	No	No	Raster	Yes	Yes	Yes	Doak *et al.* (2018 ▸)
HARE	Customized	No	No	Aperture aligned	Yes	No	Yes	Meherabi *et al.* (2020 ▸)
XTalTool	SPINE	Yes	No	Raster	Yes	Yes	No	Feiler *et al.* (2019 ▸)
MiTeGen	SPINE	Yes	No	Raster	Yes	Yes	Yes	Illava *et al.* (2021 ▸)
MISP	Customized	No	No	Aperture Aligned	Yes	No	Yes	Carrillo *et al.* (2023 ▸)
Silson	SPINE	Yes	Yes	Raster	Yes	No	No	https://silson.com/product/silicon-nitride-membranes/
**Serial-FiX**	**SPINE**	**Yes**	**Yes**	**Raster**	**Yes**	**Yes**	**No**	**This work**

**Table 2:** X-ray diffraction data-collection and refinement statistics Values in parentheses are those of the highest-resolution shell.

	Lysozyme	Lysozyme	RC_*vir*_	RC_*vir*_	*Tt* C*c*O
PDB code	9tbl	9uyr	9vdx	9s39	9vdj
Chip design	96 well	96 well	24 well	96 well	96 well
On-chip crystallization	No	Yes	Yes	No	No
Crystal type	Micro-crystals	Single crystal	Micro-crystals	Micro-crystals	LCP micro-crystals
Method	SSX	Rotational	SSX	SSX	SSX

Data collection					
Beamline	MAX IV–BioMAX	PF–AR-NW12A	MAX IV–BioMAX	ESRF–ID29	MAX IV–BioMAX
Collection temperature (K)	100	293	293	293	293
Space group	*P*4_3_2_1_2	*P*4_3_2_1_2	*P*4_3_2_1_2	*P*4_3_2_1_2	*C*2
Cell dimensions					
*a*, *b*, *c* (Å)	77.2, 77.2, 37.5	79.1, 79.1, 37.9	223.8, 223.8, 113.5	226.5, 226.5, 113.9	145.8, 100.3, 96.6
α, β, γ (°)	90, 90, 90	90, 90, 90	90, 90, 90	90, 90, 90	90, 126.8, 90
Resolution (Å)	22.1–1.75	39.6–1.6	37.0–2.8	113.25–3.0	37.2–2.3
*R*_split_ (%)†	4.8 (28)	4.9 (67.8)	21.6 (82.9)	15.7 (239.8)	23.2 (54.7)
*I*/σ(*I*)	19.9 (3.6)	16.0 (2.1)	4.1 (1.0)	5.7 (0.5)	3.07 (1.5)
CC(1/2)	99.7 (83.6)	99.9 (62.4)	93.6 (52.9)	99.9 (43.4)	85.3 (57.0)
Completeness (%)	100	95.7	100	100	99.9
Multiplicity	339	6.0	412	2142	2.0
No. collected images	95528	800 (one crystal)	150855	201910	32613
No. indexed patterns	21524	–	36048	78801	5852
No. total reflections	4050119	95667	62309072	14927547	97482
No. unique reflections	11932	16028	151216	59691	49573

Refinement					
Resolution	22.1–1.75	31.4–1.6	36.8–2.8	113.25–3.0	32.15–2.3
*R*_work_/*R*_free_ (%)	16.5/21.1	17.0/20.0	21.5/25.3	24.6/29.6	21.0/26.5
No. atoms	2064	1073	10352	10035	6429
Average *B* factor (Å^2^)	32	27.4	51.5	100.0	46.7
R.m.s deviations					
Bond lengths (Å)	0.019	0.005	0.015	0.014	0.010
Bond angles (°)	1.89	0.77	1.95	2.28	1.03

†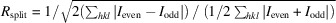.
